# Triptan efficacy does not predict onabotulinumtoxinA efficacy but improves with onabotulinumtoxinA response in chronic migraine patients

**DOI:** 10.1038/s41598-020-68149-1

**Published:** 2020-07-09

**Authors:** Ozan E. Eren, Charly Gaul, Andreas Peikert, Astrid Gendolla, Ruth Ruscheweyh, Andreas Straube

**Affiliations:** 1 Department of Neurology, Klinikum Großhadern, Ludwig Maximilians University Munich, Marchioninistr. 15, 81377 Munich, Germany; 2 Migraine and Headache Clinic Königstein, Königstein im Taunus, Germany; 3 Neurologicum Bremen, Bremen, Germany; 4 Medical Practice for Neurology and Pain Therapy Essen, Essen, Germany

**Keywords:** Headache, Migraine

## Abstract

Chronic migraine (CM) is a highly disabling primary headache. Botulinum toxin (onabotulinumtoxinA) is effective for treatment of CM, with ~ 50% of patients responding after 24 weeks. A response predictor would prevent unnecessary treatments. Inhibiting calcitonin gene related peptide (CGRP) release from trigeminal nociceptive fibres is one of the modes of acting discussed for onabotulinumtoxinA in CM. Therefore, we hypothesized that the response to triptans might predict response to onabotulinumtoxinA. Contrariwise, onabotulinumtoxinA treatment might affect triptan efficacy. 49 CM patients scheduled for their first onabotulinumtoxinA treatment were included. Before (T0) and three months after (T1) onabotulinumtoxinA treatment, patients rated triptan efficacy and indicated number of headache days/month. At T1, patients additionally rated onabotulinumtoxinA efficacy. Headache days/month were on average reduced by 7.1 ± 7.0 days from T0 to T1 (p < 0.001). Triptan efficacy ratings at T0 did not predict onabotulinumtoxinA efficacy ratings at T1 (p = 0.19) or reduction of headache days (p = 0.37). However, triptan efficacy significantly improved from T0 to T1 in onabotulinumtoxinA responders (p < 0.001) but not in non-responders (p = 1.00). Triptan efficacy did not predict response to onabotulinumtoxinA in CM. However, triptan efficacy increased after successful onabotulinumtoxinA treatment. This supports the hypothesis that efficacy of acute migraine treatment with triptans improves with effective migraine prophylaxis.

## Introduction

Chronic migraine is a highly disabling primary headache disorder recognized as a complication of migraine. Patients suffering from chronic migraine experience headache on ≥ 15 headache days/month for ≥ 3 months of which ≥ 8 headache days/month meet the criteria of migraine or are relieved by migraine specific treatment, e.g. triptans^1^. Approximately 1.4–2.2% of the general population suffers from chronic migraine^2^, with increased negative impact on quality of life and work, resulting in greater economic burden compared to episodic migraine^3–5^.

One of the substances used for preventative treatment of chronic migraine is onabotulinumtoxinA, which demonstrated a significant reduction of 8.4 headache days per month in the verum compared to the placebo group (6.6 days) after 24 weeks and two treatments. However, only about 47% of the patients respond to treatment with onabotulinumtoxinA with an improvement of ≥ 50% in headache days after 24 weeks^6^. Up to now no predictors of an overall or delayed response on onabotulinumtoxinA have been identified for use in clinical routine.

The mode of action of onabotulinumtoxinA in chronic migraine is not completely understood. Most likely, onabotulinumtoxinA is taken up by cutaneous collaterals of dural trigeminal nociceptive fibres, and after that transported^7^ to the central terminals. There, onabotulinumtoxinA reduces the release of nociceptive neurotransmitters including calcitonin gene related peptide (CGRP), substance P and glutamate. CGRP release from trigeminal fibres is known to play a key role in migraine pathophysiology^8,9^. Increased CGRP levels have been identified in jugular blood as well as in tears fluid between and during migraine attacks^10^. Therefore, patients having more release of CGRP during their migraine attacks might react better to onabotulinumtoxinA treatment. Indeed, it has been reported that chronic migraine patients have increased CGRP levels in peripheral blood, and more elevated CGRP levels are related to a better onabotulinumtoxinA response^11,12^. However, detection of CGRP from peripheral blood is difficult, especially in view of the short half-life of the peptide, making this parameter less suitable as a clinically useful predictor^10^.

Triptans, which are the most effective acute migraine treatments available, also exert part of their action by reducing CGRP release from trigeminal afferents, via agonistic action at 5-HT-1D receptors^13^. Therefore, triptans as well as onabotulinumtoxinA act by influencing the release of CGRP from trigeminal nerve fibres, we hypothesized that a good individual response to triptans in the acute migraine attack might predict a good response to onabotulinumtoxinA in the preventative treatment of chronic migraine. In order to test this hypothesis, we conducted a prospective study in chronic migraine patients scheduled for their first onabotulinumtoxinA treatment. Before (T0) and three months after (T1) onabotulinumtoxinA treatment, patients rated triptan efficacy and indicated their number of headache days/month. At T1, patients additionally rated onabotulinumtoxinA efficacy. Then, the relation between triptan and onabotulinumtoxinA efficacy was tested.

## Material and methods

The study was conducted among patients during their standard of care treatment at three different locations in Germany (Department of Neurology at the Ludwig-Maximilians-University Munich, the Migraine and Headache clinic in Königstein and a private practice specialized in headache treatment in Essen). The study was conducted in accordance with the Declaration of Helsinki and approved by the ethic committee of the medical faculty of the Ludwig-Maximillian University (340-14). All patients gave written informed consent before participation.

### Study design

Chronic migraine patients retrospectively indicated their headache frequency and triptan efficacy (see below) on the day of their first treatment with onabotulinumtoxinA (baseline, “T0”). As recommended in onabotulinumtoxinA treatment, follow-up was scheduled at 3 months. At follow-up (T1), patients answered the same questions, and additionally rated the efficacy of onabotulinumtoxinA treatment.

### Subjects

All patients were interviewed by a headache specialist and had a neurological examination. Patients with a diagnosis of chronic migraine in accordance with the IHCD-III beta^14^ criteria who at least occasionally used triptans for attack treatment and were scheduled for their first treatment with onabotulinumtoxinA (155 units) following the PREEMPT scheme^6^ were asked for study participation. An ongoing migraine preventive medication was allowed if stable for at least 3 months before participation and throughout the study. A total of 58 patients were recruited. 9 patients had to be excluded because they did not show up at the follow-up visit. This left 49 patients for analysis.

### Data acquisition

At T0, patients provided written information regarding age, sex, duration of migraine in years, number of headache days in the previous month and days with triptan use in the previous month. In addition, they rated the efficacy of triptans to treat their acute attacks (1 = very good, 2 = good, 3 = moderate, 4 = none) and the chance of being pain-free 2 h after triptan intake (1 = very good, 2 = good, 3 = moderate, 4 = none). At follow-up (T1) patients also rated the efficacy of onabotulinumtoxinA to reduce their headaches (1 = very good, 2 = good, 3 = moderate, 4 = none).

The triptans used by the patients in the present study were: rizatriptan (n = 18), zolmitriptan (n = 9), sumatriptan (n = 9), eletriptan (n = 5), naratriptan (n = 4), almotriptan (n = 3), and frovatriptan (n = 1). 37 of the 49 patients also used non-steroidal anti-inflammatory drugs (NSAIDs, n = 33) or simple analgesics (n = 4). This was not analyzed further because we were primarily interested in the relation between triptan efficacy and onabotulinumtoxinA efficacy.

Results of the chance of being pain-free 2 h after triptan intake were highly correlated with triptan efficacy ratings both at T1 (Spearman’s rho = 0.68, p < 0.001) and T2 (rho = 0.68, p < 0.001), and results were equivalent to those obtained with triptan efficacy ratings. Therefore, although we report on both for consistency, the focus will be on triptan efficacy ratings.

### Power analysis

Our primary hypothesis was that chronic migraine patients who report higher efficacy of triptans for acute migraine treatment would have a better response to onabotulinumtoxinA. Power analysis performed with G*Power^15^ indicated that using a one-way analysis of variance (ANOVA) with onabotulinumtoxinA efficacy as dependent variable and triptan efficacy at T0 as factor with four levels, a sample size of n = 48 is sufficient to detect a large effect (f = 0.50) at p < 0.05 with a power of 0.80.

### Statistical analysis

Statistical analysis was done using SPSS 23.0 (IBM Corp. Released 2015. IBM SPSS Statistics for Windows, Version 23.0. Armonk, NY: IBM Corp.). Data are given as mean ± SD or as frequencies, where adequate. Non-parametric tests were used because the main variables (efficacy of triptan and onabotulinumtoxinA) were ordinal. Wilcoxon’s test was used to compare variables before and after onabotulinumtoxinA treatment. Kruskal–Wallis ANOVA was used to compare variables between more than two groups (e.g. within the four groups of onabotulinumtoxinA efficacy). Spearman’s rho was used to test for correlations. Results were considered statistically significant at p < 0.05.

### Ethics approval and consent to participate

The study was conducted in accordance with the Declaration of Helsinki and approved by the ethics committee of the medical faculty of the Ludwig-Maximilians-University Munich (No. 340-14) and all patients gave their written informed consent. *Contact* “Ethikkommission bei der Medizinischen Fakultät der Ludwig-Maximilians-Universität München”; Pettenkoferstr. 8, 80336 Munich, Tel.: + 49 89 4400 55190; email: ethikkommission@med.uni-muenchen.de.

## Results

A total of 49 patients with chronic migraine scheduled for their first onabotulinumtoxinA treatment were included (45 women, 4 men; age 43.7 ± 12.9, duration of migraine 23.4 ± 12.3 years). At baseline (T0), patients indicated an average of 24.1 ± 5.6 headache days per month (Table 1). This was significantly reduced to 17.0 ± 8.2 days/months 3 months after treatment with onabotulinumtoxinA (T1). The average difference in headache days from T0 to T1 was 7.1 ± 7.0, corresponding to an average percent reduction in headache days per month of 30.0 ± 27.2%. The correlation between percent reduction in headache days per month and onabotulinumtoxinA efficacy ratings (see Table 1) was rho = − 0.45, p = 0.002. Inspection of the correlation showed several patients who, despite experiencing no or only a minor reduction in headache days per month, rated onabotulinumtoxinA efficacy as good or very good.

**Table 1:** Headache parameters and treatment ratings before (T0) and 3 months after (T1) treatment with onabotulinumtoxinA.

N = 49	T0	T1	Wilcoxon Z	p
Headache days/month	24.1 ± 5.6	17.0 ± 8.2	− 4.92	< 0.001
Triptan days/month	11.5 ± 6.8	6.7 ± 5.5	− 2.75	0.006
Triptan efficacy rating	2.1 ± 0.8 (11/26/9/3)	1.7 ± 0.8 (22/20/5/2)	− 3.15	0.002
Pain-free at 2 h after intake of triptan	2.1 ± 1.1 (18/17/6/8)	1.6 ± 0.8 (27/17/3/2)	− 2.81	0.005
OnabotulinumtoxinA efficacy rating	–	2.1 ± 1.0 (16/16/12/5)	–	–

Headache and triptan days/month are given as mean ± SD. For ease of interpretation, triptan and onabotulinumtoxinA efficacy ratings are also given as mean ± SD (on the scale from 1 = very good, 2 = good, 3 = moderate and 4 = none), in addition to the frequencies in the four categories.

We tested if triptan efficacy predicted onabotulinumtoxinA efficacy. There was no significant relation between triptan efficacy ratings at T0 and onabotulinumtoxinA efficacy ratings at T1 (Kruskal Wallis H = 4.83, p = 0.19, Spearman’s rho = 0.20, p = 0.16) or percent reduction of headache days from T0 to T1 (H = 3.18, p = 0.37, rho = − 0.16, p = 0.29). Therefore, there was no evidence that triptan efficacy predicted the effect of onabotulinumtoxinA in preventive treatment of chronic migraine after 3 months.

**Figure 1:**
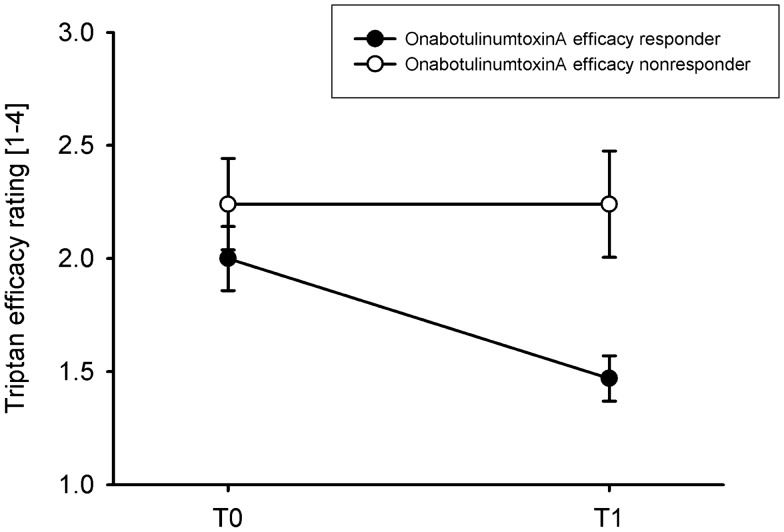
Triptan efficacy rating in onabotulinumtoxinA efficacy responders (onabotulinumtoxinA efficacy rating: very good or good, n = 32) vs. non-responders (efficacy rating: moderate or none, n = 17) is given as mean ± SEM (on the scale from 1 = very good, 2 = good, 3 = moderate and 4 = none). *T0* baseline, *T1* 3 months after onabotulinumtoxinA treatment.

Triptan efficacy ratings were significantly increased from T0 to T1 (Table 1). To further explore this relation, patients were classified as onabotulinumtoxinA efficacy responders if they had rated efficacy as very good or good (n = 32) and as non-responders if they had rated efficacy as moderate or none (n = 17). Figure 1 shows that onabotulinumtoxinA improved triptan efficacy ratings from baseline to follow-up in efficacy responders (2.0 ± 0.8 vs. 1.5 ± 0.6, Wilcoxon Z = − 3.37, p < 0.001), while triptan efficacy ratings remained unchanged in efficacy non-responders (2.2 ± 0.8 vs. 2.2 ± 1.0, Z = 0, p = 1.00). The effect was less pronounced, but still significant when response was defined according to > 30% improvement in headache days (headache day responders: n = 19; non-responders: n = 26, missing: n = 4). The triptan efficacy ratings significantly improved in headache day responders (1.9 ± 0.6 vs. 1.4 ± 0.5, Z = − 2.31, p = 0.021) but not in headache day non-responders (2.3 ± 0.9 vs. 2.0 ± 0.9, Z = − 1.94, p = 0.052).

This relationship also entailed a significant relation between triptan efficacy ratings at T1 and onabotulinumtoxinA efficacy ratings at T1 (Kruskal–Wallis H = 15.13, p < 0.001, Spearman’s rho = 0.54, p < 0.001). The percent reduction in headache days from T0 to T1 was again less strongly related to triptan efficacy ratings at T1 (H = 7.13, p = 0.068, rho = − 0.38, p = 0.009).

In conclusion, the present results suggest that triptan efficacy improved after onabotulinumtoxinA treatment in patients with a good response to onabotulinumtoxinA.

## Discussion

There are two main results to discuss.

1. The efficacy of triptans for treatment of acute migraine attacks before the first onabotulinumtoxinA treatment does not predict the efficacy of onabotulinumtoxinA for the preventive treatment of chronic migraine.
2. There was an improvement in triptan efficacy in patients with a good response to the prophylaxis with onabotulinumtoxinA.

Preventative treatment with onabotulinumtoxinA is often one of the later steps in the treatment algorithm for chronic migraine patients, and only part of the patients respond. Therefore, having a reliable predictor of response to onabotulinumtoxinA would be useful. In the present study we tested the hypothesis that a good effect of triptans in acute migraine therapy might predict a good effect of onabotulinumtoxinA in migraine prevention, because of their common action on the release of CGRP from trigeminal nerve fibres, as detailed in the introduction. However, we could find no relation between triptan efficacy before onabotulinumtoxinA treatment and response to onabotulinumtoxinA treatment, despite using two different measures to quantify the response to onabotulinumtoxinA (efficacy rating and reduction of headache days per month). To the best of our knowledge, there is only one previous report addressing this question^16^, including 44 chronic migraine patients after four cycles of onabotulinumtoxinA. Both onabotulinumtoxinA and triptan efficacy were graded at three levels (good, moderate, none). There was a significant relation between onabotulinumtoxinA response and triptan response. However, triptan response probably was assessed after the four cycles of onabotulinumtoxinA. Our results show that triptan efficacy changes with onabotulinumtoxinA treatment, and indeed triptan efficacy after onabotulinumtoxinA treatment was correlated with onabotulinumtoxinA efficacy also in our data. Therefore, results of the study cited above are consistent with our results, but in view of the change of triptan efficacy with onabotulinumtoxinA treatment can probably not be taken as indication for a prediction of onabotulinumtoxinA efficacy by triptan efficacy.

Our negative results regarding triptan response previous to treatment with onabotulinumtoxinA as a predictor might be due to the complex pathophysiology of migraine. Both onabotulinumtoxinA and triptans have several mechanisms of action, inhibition of CGRP release being only one of them^17–19^. There may be subgroups of migraine patients where CGRP plays a prominent role (and who might show a relationship between triptan and onabotulinumtoxinA efficacy), while different mechanisms or neuropeptides are implied in other patients. Therefore, in future studies we should evaluate not only the clinical characteristics but also different neuropeptides before and after onabotulinumtoxinA application to define different chronic migraine subpopulations and gain a better understanding of the underlying pathophysiology. Similar considerations also apply to the group of CGRP-(receptor)-antibodies, since in average only 30–60% of migraine patients respond to these new therapies^20^.

Interestingly, at 3 months after onabotulinumtoxinA treatment, patients with a good response to onabotulinumtoxinA also had improved their response to triptans. Improved effect of acute headache medication is one of the often cited goals of preventative migraine treatment^21^. However, there are little previous data to support that this actually happens. One small study (n = 19) reported better 2 h acute pain medication responses in onabotulinumtoxinA responders compared to saline non-responders^17^. In fact, it would also have been conceivable that triptans act less well after onabotulinumtoxinA treatment, given that both partially act by inhibiting CGRP release. Interestingly, while improvement of triptan efficacy was strong in responders and null in non-responders when subjective onabotulinumtoxinA efficacy ratings were used to define responders and non-responders, the separation between groups was less clear when using percent reduction of headache days per month. Indeed, the correlation between onabotulinumtoxinA efficacy ratings and percent reduction in headache days was only moderate (rho = − 0.45). This corroborates the clinical experience that reduction in headache days is not the only relevant parameter in assessing success of migraine prevention. There are significant numbers of patients who indicate a clinically meaningful overall improvement in their migraine (e.g. regarding headache intensity, or the ability to work), but not in the number of headache days per month.

Our study has strength and limitations. An important strength is the multicenter, prospective design. A potential limitation is the short follow-up period of 3 months. In addition, not all patients may have used a headache calendar to indicate their number of headache days per month. Furthermore, the sample size of the present study was limited, precluding the detection of small effects. However, effect sizes of the (non-significant) relation between triptan efficacy at T0 and onabotulinumtoxinA efficacy at T1 were very small (rho = 0.20), and effects of this size would not be deemed clinically significant or useful for prediction of treatment response. Furthermore, it would be interesting to have measurements of the CGRP levels before and after onabotulinumtoxinA treatment as well as during a migraine attack treated with a triptan, to better interpret the present results with respect to CGRP levels.

## Conclusions

Contrary to our hypothesis, the efficacy of triptans before onabotulinumtoxinA treatment did not predict the response to onabotulinumtoxinA treatment. However, there was an increase in triptan efficacy after onabotulinumtoxinA treatment, limited to patients with a good response to onabotulinumtoxinA. This provides evidence that an effective migraine prophylaxis also improves efficacy of acute migraine treatment.

### Key findings

- Response to triptans does not predict response to onabotulinumtoxinA in the treatment of chronic migraine.
- Triptan response improves after onabotulinumtoxinA treatment, especially in onabotulinumtoxinA responders.

## Data Availability

The datasets used and/or analyzed during the current study are available from the corresponding author on reasonable request.

## Abbreviations

CGRP: Calcitonin gene-related peptide
IHCD: International Classification of Headache Disorders 3rd edition
PREEMPT: Phase 3 REsearch Evaluating Migraine Prophylaxis Therapy
